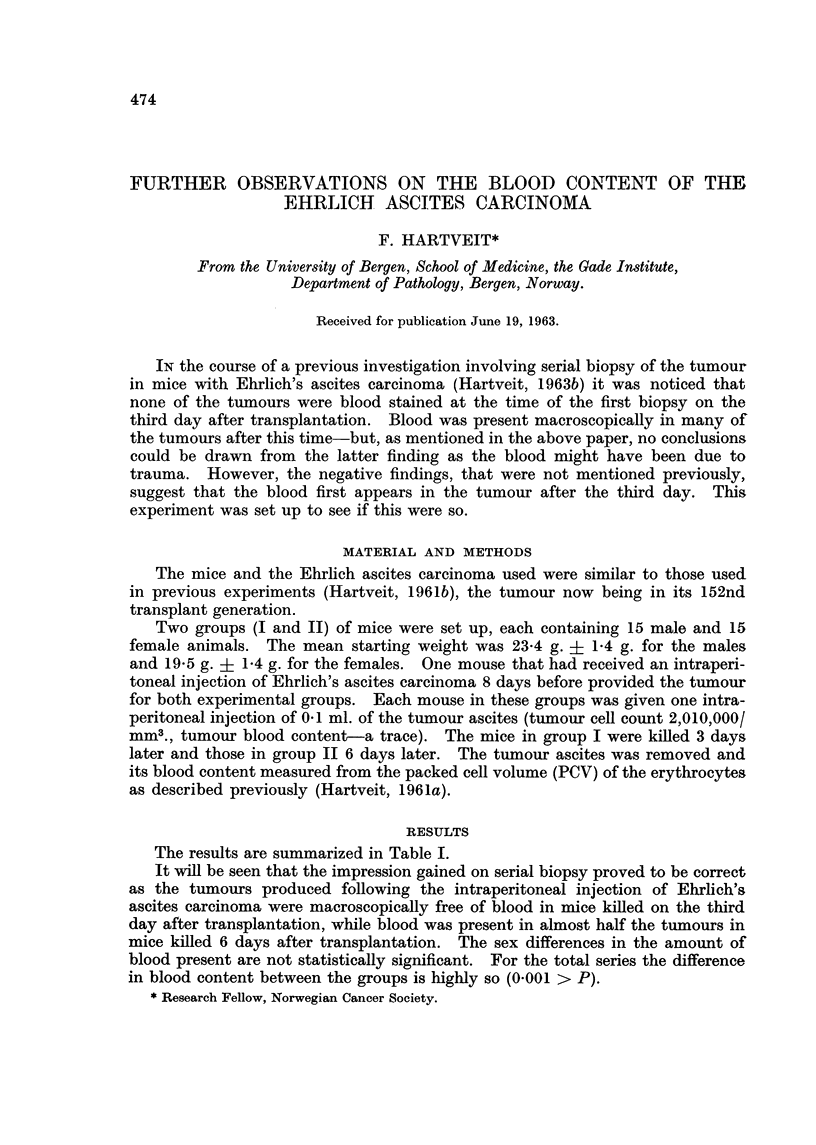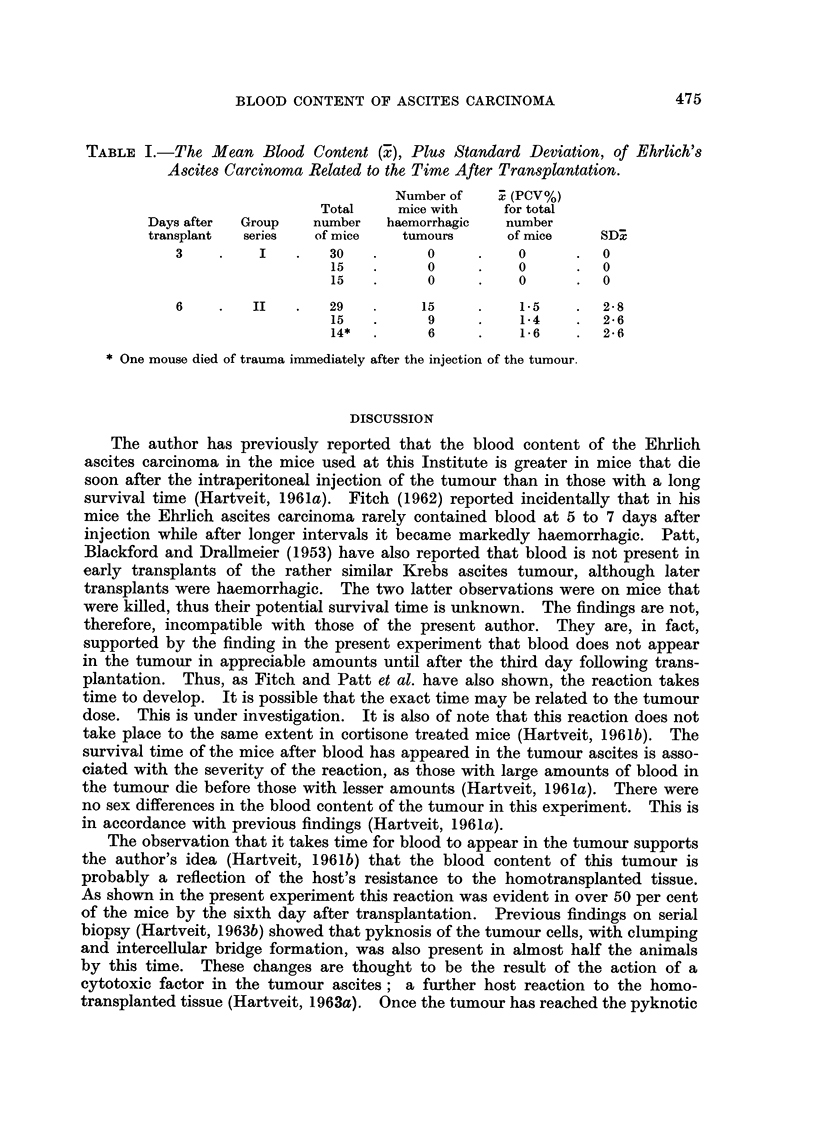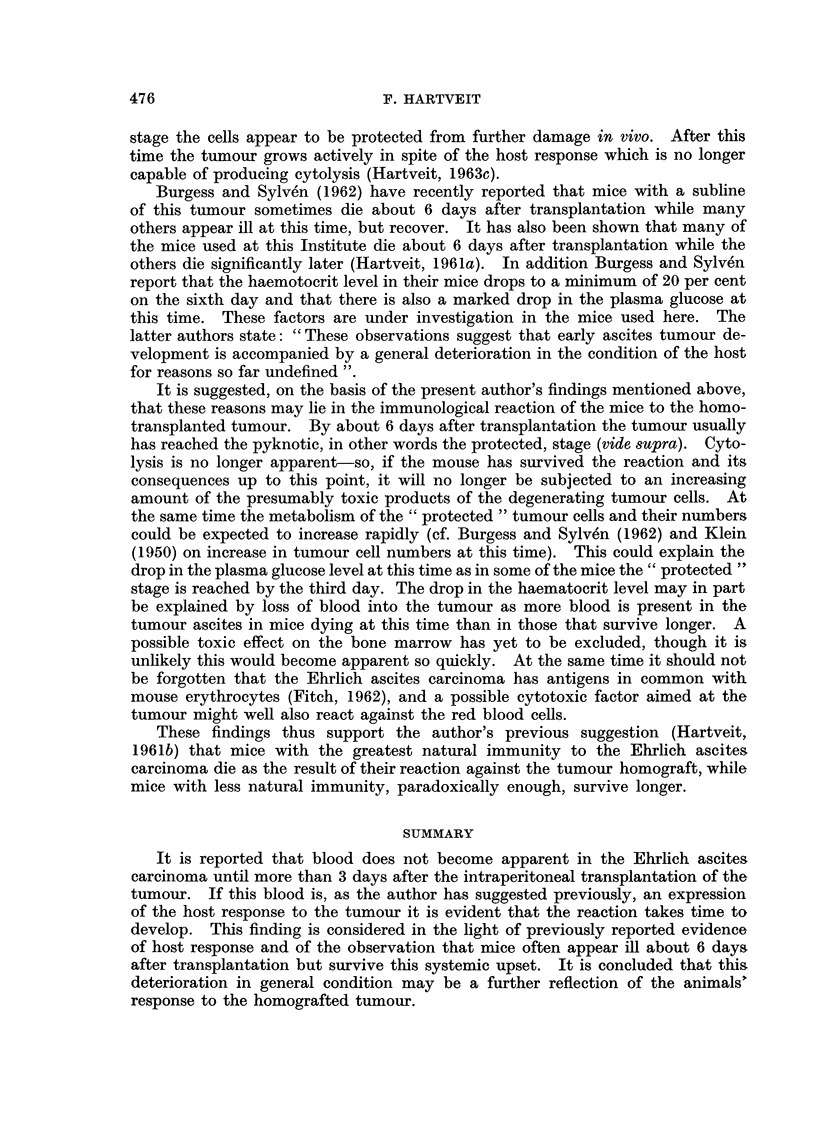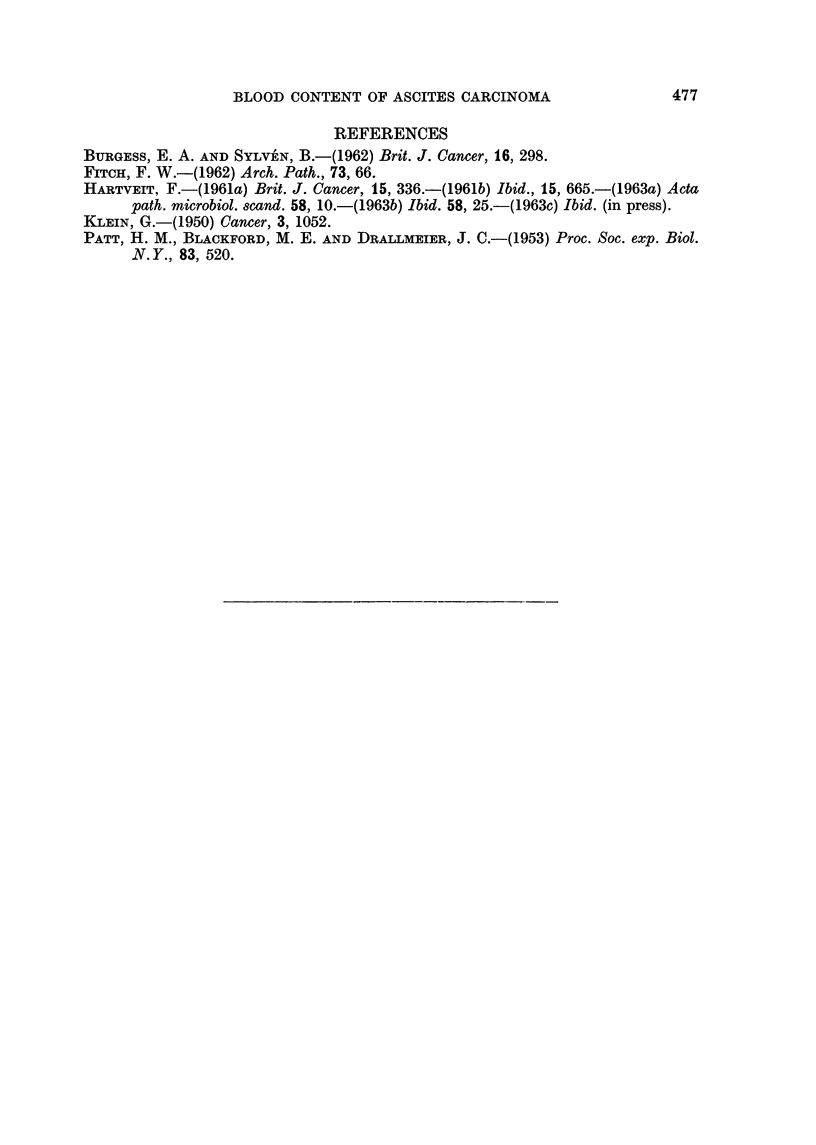# Further Observations on the Blood Content of the Ehrlich Ascites Carcinoma

**DOI:** 10.1038/bjc.1963.63

**Published:** 1963-09

**Authors:** F. Hartveit


### FURTHER OBSERVATIONS ON THE BLOOD CONTENT OF THE EHRLICH ASCITES CARCINOMA

F. HARTVEIT*

*From the University of Bergen, School of Medicine, the Gade Institute, Department of Pathology, Bergen, Norway.*

Received for publication June 19, 1963.

IN the course of a previous investigation involving serial biopsy of the tumour in mice with Ehrlich's ascites carcinoma (Hartveit, 1963*b*) it was noticed that none of the tumours were blood stained at the time of the first biopsy on the third day after transplantation. Blood was present macroscopically in many of the tumours after this time—but, as mentioned in the above paper, no conclusions could be drawn from the latter finding as the blood might have been due to trauma. However, the negative findings, that were not mentioned previously, suggest that the blood first appears in the tumour after the third day. This experiment was set up to see if this were so.

### MATERIAL AND METHODS

The mice and the Ehrlich ascites carcinoma used were similar to those used in previous experiments (Hartveit, 1961*b*), the tumour now being in its 152nd transplant generation.

Two groups (I and II) of mice were set up, each containing 15 male and 15 female animals. The mean starting weight was 23·4 g. $\pm$ 1·4 g. for the males and 19·5 g. $\pm$ 1·4 g. for the females. One mouse that had received an intraperitoneal injection of Ehrlich's ascites carcinoma 8 days before provided the tumour for both experimental groups. Each mouse in these groups was given one intraperitoneal injection of 0·1 ml. of the tumour ascites (tumour cell count 2,010,000/mm$^3$., tumour blood content—a trace). The mice in group I were killed 3 days later and those in group II 6 days later. The tumour ascites was removed and its blood content measured from the packed cell volume (PCV) of the erythrocytes as described previously (Hartveit, 1961*a*).

### RESULTS

The results are summarized in Table I.

It will be seen that the impression gained on serial biopsy proved to be correct as the tumours produced following the intraperitoneal injection of Ehrlich's ascites carcinoma were macroscopically free of blood in mice killed on the third day after transplantation, while blood was present in almost half the tumours in mice killed 6 days after transplantation. The sex differences in the amount of blood present are not statistically significant. For the total series the difference in blood content between the groups is highly so ($0{\cdot}001 > P$).

* Research Fellow, Norwegian Cancer Society.

TABLE I.—*The Mean Blood Content* ($\bar{x}$), *Plus Standard Deviation, of Ehrlich's Ascites Carcinoma Related to the Time After Transplantation.*

| Days after transplant | Group series | Total number of mice | Number of mice with haemorrhagic tumours | $\bar{x}$ (PCV%) for total number of mice | SD$\bar{x}$ |
|---|---|---|---|---|---|
| 3 | I | 30 | 0 | 0 | 0 |
| | | 15 | 0 | 0 | 0 |
| | | 15 | 0 | 0 | 0 |
| 6 | II | 29 | 15 | 1·5 | 2·8 |
| | | 15 | 9 | 1·4 | 2·6 |
| | | 14* | 6 | 1·6 | 2·6 |

* One mouse died of trauma immediately after the injection of the tumour.

### DISCUSSION

The author has previously reported that the blood content of the Ehrlich ascites carcinoma in the mice used at this Institute is greater in mice that die soon after the intraperitoneal injection of the tumour than in those with a long survival time (Hartveit, 1961*a*). Fitch (1962) reported incidentally that in his mice the Ehrlich ascites carcinoma rarely contained blood at 5 to 7 days after injection while after longer intervals it became markedly haemorrhagic. Patt, Blackford and Drallmeier (1953) have also reported that blood is not present in early transplants of the rather similar Krebs ascites tumour, although later transplants were haemorrhagic. The two latter observations were on mice that were killed, thus their potential survival time is unknown. The findings are not, therefore, incompatible with those of the present author. They are, in fact, supported by the finding in the present experiment that blood does not appear in the tumour in appreciable amounts until after the third day following transplantation. Thus, as Fitch and Patt *et al.* have also shown, the reaction takes time to develop. It is possible that the exact time may be related to the tumour dose. This is under investigation. It is also of note that this reaction does not take place to the same extent in cortisone treated mice (Hartveit, 1961*b*). The survival time of the mice after blood has appeared in the tumour ascites is associated with the severity of the reaction, as those with large amounts of blood in the tumour die before those with lesser amounts (Hartveit, 1961*a*). There were no sex differences in the blood content of the tumour in this experiment. This is in accordance with previous findings (Hartveit, 1961*a*).

The observation that it takes time for blood to appear in the tumour supports the author's idea (Hartveit, 1961*b*) that the blood content of this tumour is probably a reflection of the host's resistance to the homotransplanted tissue. As shown in the present experiment this reaction was evident in over 50 per cent of the mice by the sixth day after transplantation. Previous findings on serial biopsy (Hartveit, 1963*b*) showed that pyknosis of the tumour cells, with clumping and intercellular bridge formation, was also present in almost half the animals by this time. These changes are thought to be the result of the action of a cytotoxic factor in the tumour ascites ; a further host reaction to the homotransplanted tissue (Hartveit, 1963*a*). Once the tumour has reached the pyknotic stage the cells appear to be protected from further damage *in vivo*. After this time the tumour grows actively in spite of the host response which is no longer capable of producing cytolysis (Hartveit, 1963*c*).

Burgess and Sylvén (1962) have recently reported that mice with a subline of this tumour sometimes die about 6 days after transplantation while many others appear ill at this time, but recover. It has also been shown that many of the mice used at this Institute die about 6 days after transplantation while the others die significantly later (Hartveit, 1961*a*). In addition Burgess and Sylvén report that the haemotocrit level in their mice drops to a minimum of 20 per cent on the sixth day and that there is also a marked drop in the plasma glucose at this time. These factors are under investigation in the mice used here. The latter authors state: "These observations suggest that early ascites tumour development is accompanied by a general deterioration in the condition of the host for reasons so far undefined ".

It is suggested, on the basis of the present author's findings mentioned above, that these reasons may lie in the immunological reaction of the mice to the homotransplanted tumour. By about 6 days after transplantation the tumour usually has reached the pyknotic, in other words the protected, stage (*vide supra*). Cytolysis is no longer apparent—so, if the mouse has survived the reaction and its consequences up to this point, it will no longer be subjected to an increasing amount of the presumably toxic products of the degenerating tumour cells. At the same time the metabolism of the " protected " tumour cells and their numbers could be expected to increase rapidly (cf. Burgess and Sylvén (1962) and Klein (1950) on increase in tumour cell numbers at this time). This could explain the drop in the plasma glucose level at this time as in some of the mice the " protected " stage is reached by the third day. The drop in the haematocrit level may in part be explained by loss of blood into the tumour as more blood is present in the tumour ascites in mice dying at this time than in those that survive longer. A possible toxic effect on the bone marrow has yet to be excluded, though it is unlikely this would become apparent so quickly. At the same time it should not be forgotten that the Ehrlich ascites carcinoma has antigens in common with mouse erythrocytes (Fitch, 1962), and a possible cytotoxic factor aimed at the tumour might well also react against the red blood cells.

These findings thus support the author's previous suggestion (Hartveit, 1961*b*) that mice with the greatest natural immunity to the Ehrlich ascites carcinoma die as the result of their reaction against the tumour homograft, while mice with less natural immunity, paradoxically enough, survive longer.

### SUMMARY

It is reported that blood does not become apparent in the Ehrlich ascites carcinoma until more than 3 days after the intraperitoneal transplantation of the tumour. If this blood is, as the author has suggested previously, an expression of the host response to the tumour it is evident that the reaction takes time to develop. This finding is considered in the light of previously reported evidence of host response and of the observation that mice often appear ill about 6 days after transplantation but survive this systemic upset. It is concluded that this deterioration in general condition may be a further reflection of the animals' response to the homografted tumour.

### REFERENCES

BURGESS, E. A. AND SYLVÉN, B.—(1962) *Brit. J. Cancer*, **16**, 298.
FITCH, F. W.—(1962) *Arch. Path.*, **73**, 66.
HARTVEIT, F.—(1961*a*) *Brit. J. Cancer*, **15**, 336.—(1961*b*) *Ibid.*, **15**, 665.—(1963*a*) *Acta path. microbiol. scand.* **58**, 10.—(1963*b*) *Ibid.* **58**, 25.—(1963*c*) *Ibid.* (in press).
KLEIN, G.—(1950) *Cancer*, **3**, 1052.
PATT, H. M., BLACKFORD, M. E. AND DRALLMEIER, J. C.—(1953) *Proc. Soc. exp. Biol. N.Y.*, **83**, 520.